# A process mining- deep learning approach to predict survival in a cohort of hospitalized COVID‐19 patients

**DOI:** 10.1186/s12911-022-01934-2

**Published:** 2022-07-25

**Authors:** M. Pishgar, S. Harford, J. Theis, W. Galanter, J. M. Rodríguez-Fernández, L. H Chaisson, Y. Zhang, A. Trotter, K. M. Kochendorfer, A. Boppana, H. Darabi

**Affiliations:** 1 Department of Mechanical and Industrial Engineering, University of Illinois at Chicago (UIC), 842 W Taylor Street, MC 251, Chicago, IL 60607 USA; 2 Departments of Medicine and Pharmacy Systems, Outcomes and Policy, UIC, Chicago, USA; 3 Department of Neurology, Clinical Informatics Fellowship, UIC, Chicago, USA; 4 Department of Medicine, UIC, Chicago, USA; 5 University of Illinois Hospital (UIH), UIC, Chicago, USA; 6 Department of Family and Community Medicine, UIC, Chicago, USA

**Keywords:** Mortality prediction, Process mining, Deep learning, COVID-19 prediction, Machine learning, SARS-CoV-2

## Abstract

**Background:**

Various machine learning and artificial intelligence methods have been used to predict outcomes of hospitalized COVID-19 patients. However, process mining has not yet been used for COVID-19 prediction. We developed a process mining/deep learning approach to predict mortality among COVID-19 patients and updated the prediction in 6-h intervals during the first 72 h after hospital admission.

**Methods:**

The process mining/deep learning model produced temporal information related to the variables and incorporated demographic and clinical data to predict mortality. The mortality prediction was updated in 6-h intervals during the first 72 h after hospital admission. Moreover, the performance of the model was compared with published and self-developed traditional machine learning models that did not use time as a variable. The performance was compared using the Area Under the Receiver Operator Curve (AUROC), accuracy, sensitivity, and specificity.

**Results:**

The proposed process mining/deep learning model outperformed the comparison models in almost all time intervals with a robust AUROC above 80% on a dataset that was imbalanced.

**Conclusions:**

Our proposed process mining/deep learning model performed significantly better than commonly used machine learning approaches that ignore time information. Thus, time information should be incorporated in models to predict outcomes more accurately.

## Background

Throughout the COVID-19 pandemic, machine learning and artificial intelligence (AI) methods have been used to understand and predict virus spread, the potential impact of vaccines, morbidity, mortality, and resource allocation [1]. Modeling of COVID-19 morbidity and mortality has yielded insights into disease progression [2, 3], which have been informative for health systems to anticipate resource needs and effective interventions [4]. However, with the emergence of COVID-19 variants and rapid advances in COVID-19 treatment, prevention, and vaccination, 1-time modeling is likely ineffective for understanding how to provide optimal care from the patient, health system, and public health perspectives [4].

Process mining techniques assist in analyzing and optimizing systems using sequences of observations. Process mining approaches have been shown to be valuable in the healthcare industry by enhancing healthcare processes [5, 6]. However, process mining has not yet been used to predict mortality after hospital admission for COVID-19 patients [7, 8] though providing significant advantages over static models. In general, process mining algorithms take a sequential perspective on data points that have been observed over time to derive a single semantic-rich graph structure like a Petri Net. In the context of COVID-19, each patient follows a distinct path throughout such a derived Petri net while being in one state at any point of time. The states naturally embed information of the sequence of observations that lead to this state and of potential future observations leading to subsequent states. This means that process mining algorithms allow to explicitly incorporate the timing and sequence of healthcare events into the modeling process by leveraging the states of a Petri Net.

One significant advantage of process mining techniques over static models is their ability to explicitly incorporate the timing and sequence of healthcare events into the modeling process. For example, let’s assume that a machine learning model uses two specific inputs of blood pressure and blood sugar to predict the mortality of a patient. In this case, a static machine learning model is indifferent to the sequence by which the values of blood pressure and blood sugar were obtained from the patient. Also, the model does not consider when these values were collected (the occurrence times of the events associated with collecting blood pressure and blood sugar values are ignored by the model) in predicting the mortality of the patients. In contrast, for this example, a process mining model uses not only the values of blood pressure and blood sugar, but by leveraging Petri net states, also their collection sequence, and timing in calculating the mortality of the patient. It can be shown that by incorporating the time and sequence information, one can usually generate better prediction models [9]. Therefore, we aimed to utilize a combined process mining and deep learning modeling approach for prediction.

## Methodology

### University of illinois hospital (UIH) cohort and variables

UIH is a tertiary, academic teaching hospital in Chicago. The University of Illinois at Chicago (UIC) Institutional Review Board approved this study. All admissions to UIH for COVID-19 positive patients were reviewed for the time of the first COVID-19 positive test and the date of admission. If the first positive COVID-19 test was performed greater than 14 days prior to admission or greater than 48 h after admission, the patient was excluded. Patients transferred from another institution were reviewed for prior COVID-19 testing. The patient was excluded if the most recent COVID-19 test has been performed longer than 14 days prior to the transfer. If the transfer was not related to any possible COVID-19 symptoms, the patient was excluded. Symptomatic patients for COVID-19 were included in this cohort, as verified by manual chart review or claim data.

If a patient had multiple hospital admissions at UIH related to COVID-19, each admission encounter was categorized with a final outcome of as death or discharge. All admissions were categorized as intensive care unit (ICU) or Non-ICU.

We partitioned our data into training, validation, and test cohorts using a 60/20/20 split ratio, respectively. Consequently, each admission encounter belonged to a unique cohort.

Variable selection was based on literature review and expert opinion [10]. The variables selected are shown in Table 6, in the appendix section, where demographics, vital signs, laboratory data, and clinical characteristics (comorbidities, diagnosis codes, problem list, clinic notes, procedure reports, location within the hospital) were assessed.

### Converting electronic health records (EHRs) to an event log

Process mining algorithms utilize event logs as their input. Event logs consist of a sequence of events with a name describing the observed action and its corresponding timestamp (i.e., when the event occurred). The temporally ordered sequence of such events is called a trace. Commonly, a trace contains only events that belong to the same context. In this paper, the observations of a specific COVID-19 admission formed a trace. This can also be understood as a trajectory. The set of all traces (i.e., all COVID-19 admissions in the dataset) comprised an event log.

The extracted traces of the event log were performed at 6 h, 12 h, 18 h, 24 h, 30 h, 36 h, 42 h, 48 h, 54 h, 60 h, 66 h, and 72 h of the hospital admission. Patients that had died or been discharged before a given time of the prediction were excluded from contributing date to times after discharge or death.

For each admission, static features were extracted that did not change over the course of the hospital encounter (i.e. demographic information, comorbidities). The patient-centric trajectory of the hospital encounter was then represented as a trace. A trace started with the first occurrence of an event related to the hospital encounter and ended with the occurrence of an outcome event: either discharge or death. Each event was associated with the timestamp of observation. In this way, the state of the patient can be reconstructed at each point of time. Events can be either location-based, vital signs, lab measurements, report-based, encounter-based, or ICU-based.

Location-based events represented that a patient moved to a particular location. For example: the emergency room, ICU, non-ICU inpatient teams, among others. Vital sign events represented the observation of a particular vital sign, which were subsequently recorded as either “ok” or “critical”. Laboratory measurements were flagged as either normal or abnormal to create the laboratory events. Report-based events corresponded to procedure reports (e.g. electrocardiograms or radiological testing). Report-based events correspond to a performed procedure without considering individual findings or outcomes within the reports. Encounter-based events represented specific highlights (admission, observation status, discharge, or death) during the hospital stay. ICU-based events were based on the admission or not to the ICU, therefore, there were ICU-in and ICU-out events recorded.

After the conversion of the EHR data, a set of traces (i.e., an event log) was obtained. Each set of traces corresponded to one hospital admission and used the events to describe the health trajectory of the patient from admission to either discharge or death. Due to the definition of events and the sequential structure of traces, the traces could be used to create subtraces, such that a subtrace contained only events from, e.g., admission time to 24 h into the hospital encounter.

### Process mining/deep learning model development

**Fig. 1:**
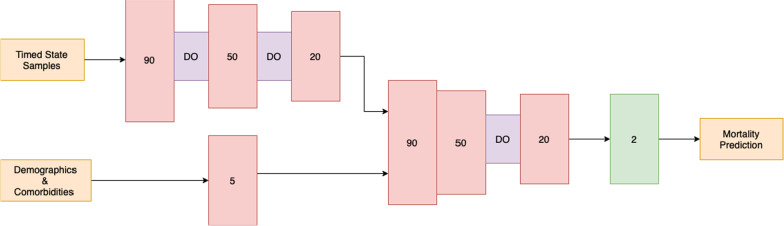
Architecture of Neural Network (NN). This Figure shows the details of the NN architecture. The timed state samples, demographics information and comorbidities were fed separately to two branches which first branch contains three hidden layers with 90, 50 and 20 neurons respectively. After the first and after the second hidden layers, there is a dropout layer with a rate of 20%. Moreover, the second branch contains one hidden layer with 5 neurons. The two branches were then concatenated to a branch with three hidden layers, containing 90, 50, and 20 neurons respectively. There is a dropout layer after the second concatenated hidden layer with the rate of 30%. At the end, the output layer included softmax activation function to predict mortality of the COVID- 19 patients

**Fig. 2:**
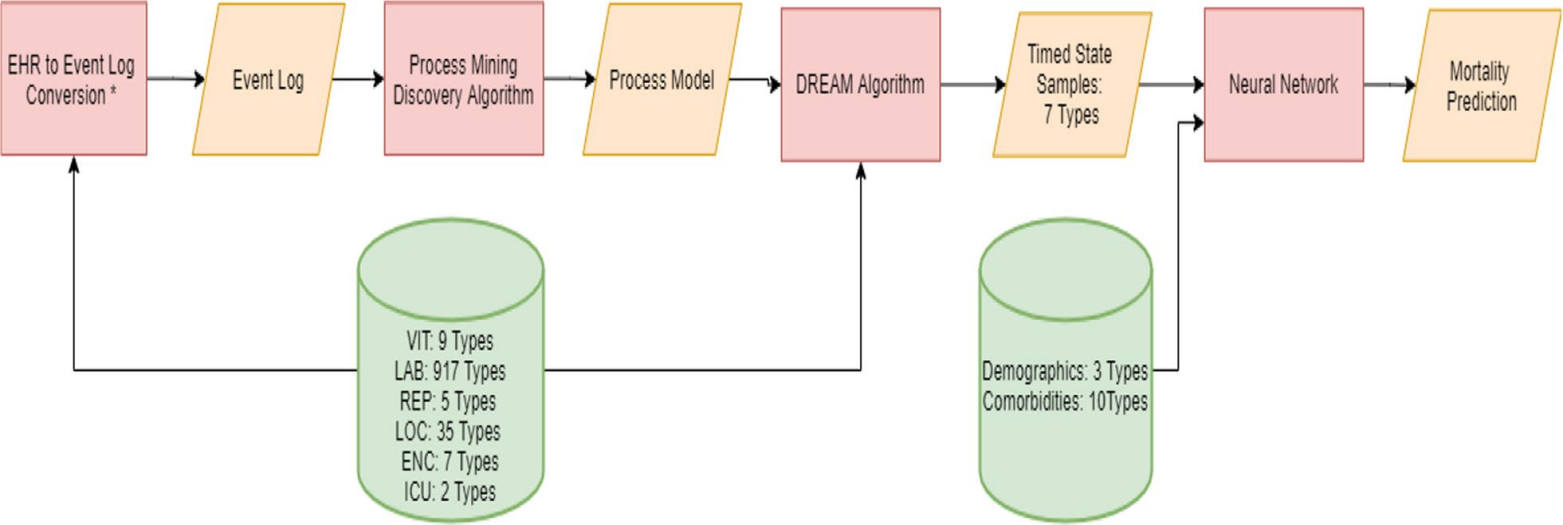
Process Mining/Deep Learning Model Development: The orange parallelograms represent the input/ output data. Four different algorithms were used in this methodology which is shown in red rectangles. The green cylinders represent the variable types that were coming directly from the database and were used as the inputs to the algorithms. *Refer to Section *Converting Electronic Health Records (EHRs) to an Event Log* for more details

A process mining/deep learning model was developed to predict the likelihood of mortality every 6-h within the first 72 h of hospital admission. Our approach is a combination of both process mining and deep learning modeling. The process mining modeling output were used as the input to the deep learning model for the prediction. The patient trajectories were used to extract a process graph model using a process mining discovery algorithm [11]. The resulting process model and the patient trajectories from admission to the time of prediction were fed to the Decay Replay Mining (DREAM) algorithm [12]. The DREAM algorithm enhances the process model with functions that parameterize time using the patient trajectories. As an output, the DREAM algorithm provides a state of the process model for each patient that contains time information. Hence, the outputs of the DREAM algorithm are called timed state samples (TSS). The TSS corresponds to the health condition of a patient up to the time of prediction and contains information on the observed events and process states, and their interarrival times. Comorbidities and demographic information were used as independent variables. The generated TSS, together with demographic information and comorbidities, were then fed to a Neural Network (NN) model to predict mortality for each 6-h interval within the first 72 h. The same process model was used for all time intervals, and the architecture of the NN is shown in Fig. 1. Also, Table 1 provides more details about the deep learning modeling parameters. Figure 2 illustrates the complete overview of our proposed approach. The corresponding source code is publicly available on our Github repository. Descriptive statistics, model development, and statistical analysis were conducted using Python, version 3.6.

**Table 1:** Deep learning model parameters

Hours	Epoch	Batch size	Dropout rate	Activation function	Learning rate	optimizer
6,12, 18, 30, 42, 54, 60, 66, 72	350	12	0.5	Relu	5e-4	Adam
24, 36	350	12	0.7	Relu	5e-4	Adam
48	350	8	0.7	Relu	5e-4	Adam

### Machine learning models

We compared the results of the process mining approach with results of a published model and self-developed models using machine learning algorithms that did not directly utilize time information.

The first model was a Logistic Regression (LR) model developed using data from 305 patients in China [13]. Core features in this model were age, Lactate dehydrogenase (LDH), and C-reactive protein (CRP).

The self-developed model was trained using the UIH data cohorts to explore other machine learning algorithms for the time interval modeling task. The development of these models utilized the variables described above. However, the data were kept in the original tabular format, as opposed to the event log format. The time component of the data was implicitly added to the training process by splitting a single training instance into multiple instances based on the time interval. This conversion allowed the developed models to witness instances from low time intervals that had limited information and from high intervals with more complete information. A variety of popular machine learning algorithms were evaluated to classify mortality at each 6- hour time interval within 72 h of admission. These algorithms included Logistic Regression (LR) [14], Decision Trees [15], Support Vector Machine (SVM) [16], Random Forest [17], XGBoost [18], LightGBM [19], and CatBoost [20]. The training process of these models included both a forward step feature selection and a grid search of model parameters. This search process aimed to find the best model with the fewest input features. The best model was determined based on the Average Area Under the Receiver Operating Characteristic Curve (AUROC) [21] of the validation cohort at each time interval.

### Model evaluation

The primary evaluation metric for model development and selection was the AUROC. We used Delong’s test to calculate 95% confidence intervals (CI) of the AUROCs and compare AUROC CIs between models [22]. In addition, we calculated the accuracy, sensitivity and specificity of models across the time intervals [22], with 95% CIs.

### Analysis of contribution of process mining unique variables

Shapley value analysis [23] was conducted on the testing cohort to find out the impact of each variable in the process mining model prediction and to identify variables associated with the mortality prediction of the COVID-19 patients in the 6-h intervals within the first 72 h, and to compare it to the self-developed machine learning and Chinese LR [13] models.

## Results

### UIH cohort characteristics

Table 2 shows the demographics, clinical characteristics, and medical conditions of the study population per encounter. There was a total of 508 encounters of 481 unique patients. The training cohort included 303 encounters (60%), the validation and testing cohorts the remaining 101 (20%) and 104 (20%) encounters, respectively. Given the size of the data, more traditional machine learning models have an advantage over deep learning based models. With the emergence of more COVID-19 data these models have the potential to be updated with more information. In the current state, data augmentation methods have the potential to be implemented with the goal of increasing overall performance. In this study, we do not implement any data augmentation, as the purpose of this work is to focus on the utilization of time information through the process mining algorithms.

**Table 2:** Encounter characteristics of the training, validation, and testing cohorts

Characteristics	Training cohort (*N* = 303)	Validation cohort (*N* = 101)	Testing cohort (*N* = 104)	*p*-value train versus Test*	*p*-value validation versus test*	*p*-value train + validation versus test*
Number of unique patients N (%)	288 (95.0)	96 (95.0)	97 (93.3)			
Primary outcome *(N, (%))*						
Mortality	43 (14.2)	6 (5.9)	11 (10.6)	**0.18**	**0.12**	** < 0.0001**
Demographics						
Age in years Mean (std)	56.6 (16.6)	56.6 (15.6)	53.4 (14.2)	**0.012**	**0.028**	**0.009**
Female N (%)	147 (48.5)	50 (49.5)	56 (53.8)	**0.18**	**0.27**	**0.18**
Race/ethnicity *(N, (%))*				**0.63**	**0.95**	**0.76**
Black	137 (45.2)	51 (50.5)	49 (47.1)			
Hispanic	36 (11.9)	13 (12.9)	16 (15.4)			
Other, non- hispanic	112 (37.0)	30 (29.7)	32 (30.7)			
White	18 (5.9)	7 (6.9)	7 (6.7)			
Mean (std) of the number of laboratory measurements per encounter						
	636 (786)	510 (663)	531 (972)	**0.078**	**0.228**	**0.090**
Mean (std) vital signs measurements per encounter						
	999 (1540)	765 (1344)	802 (1971)	**0.026**	**0.12**	**0.030**
**Comorbidities**				**0.81**	**0.69**	**0.81**
Mean (std) comorbidities per encounter	1.0 (1.1)	1.0 (1.1)	0.9 (0.9)			
Hypertension N (%)	128 (42.2)	43 (42.6)	37 (35.6)			
Diabetes N (%)	89 (29.4)	32 (31.7)	30 (28.8)			
Heart disease N (%)	12 (3.9)	1 (1.0)	2 (1.9)			
COPD N (%)	3 (1.0)	0 (0.0)	1 (1.0)			
Stroke N (%)	1 (0.3)	0 (0.0)	0 (0.0)			
Cerebrovascular disease N (%)	0 (0.0)	2 (2.0)	0 (0.0)			
Cancer N (%)	4 (1.3)	2 (2.0)	1 (1.0)			
Respiratory problems N (%)	44 (14.5)	12 (11.9)	15 (14.4)			
Chronic kidney disease N (%)	28 (9.2)	11 (10.9)	6 (5.7)			
Tuberculosis N (%)	3 (1.0)	1 (1.0)	3 (2.9)			

Bold indicates* p*-value < 0.05

Significance was set at 0.05

Patients older than 89 have been clipped to age 90

^*^Continuous variables were compared using a t-test and categorical variables were compared using a Chi-square test

The testing cohort was slightly younger than the training and validation cohorts (mean 53.4 vs. 56.6 years, *p* = 0.009). Though the distribution of race was not significantly different between the cohorts, the proportion of self-described Black patients was slightly higher in the validation (50.5%) and testing (47.1%) cohorts compared to the training cohort (45.2%). There were no statistically significant differences in the number of comorbidities per encounter in each cohort.

There were statistically more events in the training cohort (516.0 ± 3,882.3), compared to the testing (186.8 ± 1,217.4) and validation (176.6 ± 1,133.4) cohorts (*P* = 0.014). Conversely, there were no statistically significant differences across encounter types by cohort (*P* = 0.96); laboratory events were the most frequent (94%, 94%, and 93% in the training, testing, and validation cohorts, respectively), followed by location (3.6%, 3.3% and 4.3% in the training, testing and validation cohorts, respectively) and vital signs events (0.9%, 1.2% and 1.2% in the training, testing and validation cohorts, respectively).

### Evaluation metrics and proposed and baseline model performance

**Fig. 3:**
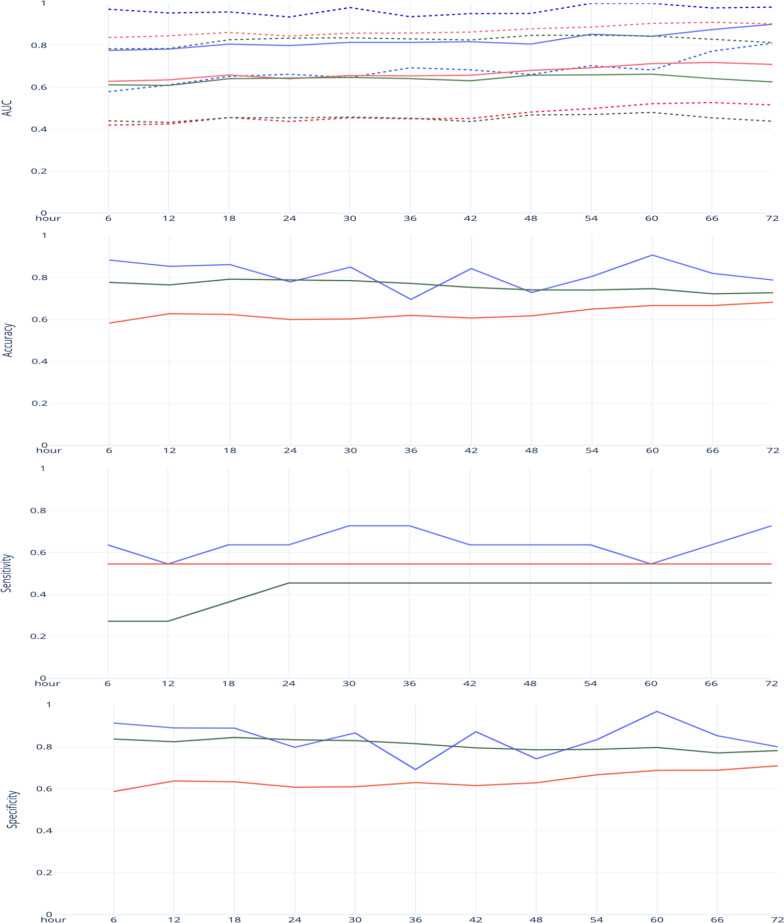
Statistical metrics for all 6-h intervals within the first 72 h on the testing cohort. Blue indicators the Process Mining Model. Green indicators the Random Forest Model. Red indicators the Logistical Regression Model. Dashed lines indicate the upper and lower 95% confidence interval of the model’s AUROC

The process mining/ deep learning approach surmounted all of the time intervals in terms of AUROC compared to both the best baseline model and the best existing model in the literature. Also, in terms of specificity and accuracy, the proposed approach yielded the highest results in 9 intervals out of 12. Lastly, comparing the sensitivity metric results, our proposed model resulted in the best results in 10 intervals. The summary of the evaluation metrics for both the proposed approach and the baseline models is illustrated in Fig. 3 (detailed numbers in Table 3). Moreover, Table 4 shows an evaluation of the sensitivity and specificity for the three models. Hence, the experimental results indicate that our approach outperformed all evaluation metrics in most time intervals. A t-test of means is performed to test the stated null and alternative hypothesis for both the sensitivity and specificity over the 72-h time range with a threshold of 0.5. This analysis shows that the PM model outperformed both the RF and LR models.

**Table 3:** Detailed results on the testing cohort

Hour	Confusion matrix			AUROC			Specificity			Sensitivity			Accuracy			
	PM	RF	LR	PM	RF	LR	PM	RF	LR	PM	RF	LR		PM	RF	LR
6	84;8 4;7	54;38 5;6	77;15 8;3	0.776	0.628	0.611	0.913	0.587	0.837	0.636	0.545	0.273		0.883	0.583	0.776
12	81;10 5;6	58;33 5;6	75;16 8;3	0.782	0.635	0.608	0.890	0.637	0.824	0.545	0.545	0.273		0.853	0.627	0.765
18	80;10 4;7	57;33 5;6	76;14 7;4	0.806	0.658	0.640	0.889	0.633	0.844	0.636	0.545	0.364		0.861	0.624	0.792
24	67;17 4;7	51;33 5;6	70;14 6;5	0.799	0.640	0.644	0.798	0.607	0.833	0.636	0.545	0.455		0.779	0.600	0.789
30	71;11 3;8	50;32 5;6	68;14 6;5	0.814	0.656	0.646	0.866	0.610	0.829	0.727	0.545	0.455		0.849	0.602	0.785
36	56;25 3;8	51;30 5;6	66;15 6;5	0.814	0.654	0.641	0.691	0.630	0.815	0.727	0.545	0.455		0.696	0.619	0.771
42	68;10 4;7	48;30 5;6	62;16 6;5	0.817	0.657	0.631	0.872	0.615	0.795	0.636	0.545	0.455		0.843	0.606	0.752
48	52;18 4;7	44;26 5;6	55;15 6;5	0.806	0.680	0.657	0.743	0.629	0.786	0.636	0.545	0.455		0.728	0.617	0.740
54	55;11 4;7	44;22 5;6	52;14 6;5	0.853	0.692	0.659	0.833	0.667	0.788	0.636	0.545	0.455		0.805	0.649	0.740
60	62;2 5;6	44;20 5;6	51;13 6;5	0.843	0.713	0.662	0.969	0.688	0.797	0.545	0.545	0.455		0.907	0.667	0.746
66	52;9 4;7	42;19 5;6	47;14 6;5	0.875	0.718	0.641	0.852	0.689	0.770	0.636	0.545	0.455		0.819	0.667	0.722
72	44;11 3;8	39;16 5;6	43;12 6;5	0.9	0.709	0.625	0.800	0.709	0.782	0.727	0.545	0.455		0.788	0.681	0.727

**Table 4:** Statistical comparison of evaluation metrics

Hypothesis		AUROC (*p*-value)
Null	Alternative	
PM = LR	PM > LR	< 0.05 (PM has a significantly better AUROC than LR)
PM = LR	LR > PM	> 0.05 (LR does not have a significantly better AUROC than PM)
PM = RF	PM > RF	< 0.05 (PM has a significantly better AUROC than RF)
PM = RF	RF > PM	> 0.05 (RF does not have a significantly better AUROC than PM)
RF = LR	RF > LR	> 0.05 (RF does not have a significantly better AUROC than LR)
RF = LR	LR > RF	> 0.05 (LR does not have a significantly better AUROC than RF)

### Shapley value analysis

**Fig. 4:**
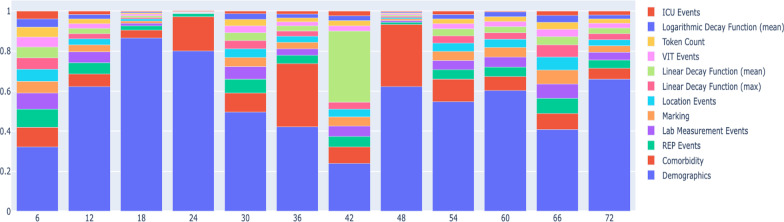
illustrates the results of the Shapley value analysis for all 6-h intervals within the first 72 h of COVD-19 patients

Figure 4 illustrates the results of the Shapley value analysis for all 6-h intervals within the first 72 h of admission. Also, the exact Shapley values are shown in Table 5. In almost all cases, demographic characteristics had the most significant impact on the prediction of mortality, followed by comorbidities. Age was strongly associated with mortality [9]. The impact of other variables varied from one time interval to another and comparing the value of the Shapley analysis for other variables, no consistent order was observed. The Shapley value analysis confirmed that the process mining-related variables–including the time decay function values, markings, and token counts– were consistently important for predicting mortality .

**Table 5:** Shapley value analysis summary

Category	Time intervals											
	6 Hr	12Hr	18Hr	24Hr	30Hr	36Hr	42Hr	48 Hr	54Hr	60Hr	66Hr	72Hr
Demographics	0.0144	0.0706	0.5983	1.014	0.0657	0.0622	0.0222	0.2034	0.0422	0.0274	0.0199	0.0698
Comorbidity	0.0044	0.0071	0.0264	0.2162	0.0126	0.0465	0.0076	0.1012	0.0087	0.0032	0.0039	0.0058
REP Events	0.0041	0.0064	0.0143	0.0201	0.0092	0.0061	0.0049	0.0041	0.0036	0.0022	0.0037	0.0044
Lab Measurement events	0.0035	0.0062	0.0092	0.0023	0.0083	0.0048	0.0048	0.0026	0.0036	0.0022	0.0035	0.0041
marking	0.0027	0.0040	0.0079	0.0023	0.0061	0.0048	0.0043	0.0025	0.0034	0.0022	0.0034	0.0033
Location events	0.0027	0.0033	0.0068	0.0023	0.0058	0.0044	0.0035	0.0023	0.0032	0.0019	0.0032	0.0033
Linear decay function (max)	0.0025	0.0030	0.0058	0.0022	0.0053	0.0039	0.0033	0.0022	0.0028	0.0015	0.0029	0.0032
Linear decay function (mean)	0.0024	0.0030	0.0055	0.0018	0.0052	0.0038	0.033	0.0022	0.0028	0.0013	0.0020	0.0029
VIT events	0.0023	0.0027	0.0053	0.0017	0.0046	0.0031	0.0025	0.0019	0.0020	0.0012	0.0018	0.0026
Token count	0.0022	0.0027	0.0044	0.0017	0.0042	0.0028	0.0023	0.0016	0.0018	0.0011	0.0017	0.0024
Logarithmic decay function (mean)	0.0018	0.0026	0.0042	0.0016	0.0038	0.0027	0.0023	0.0015	0.0017	0.0011	0.0017	0.0021
ICU Events	0.0018	0.0019	0.0026	0.0013	0.0018	0.0024	0.0022	0.0014	0.0013	0.0002	0.0011	0.0020

## Discussion

Using a cohort of hospitalized COVID-19 patients from a large medical center in the United States, we developed a process mining model using routine clinical data and the sequence of clinical events to evaluate mortality risk. Process mining performed significantly better than traditional predictive models over 6-h intervals within the first 72 h after hospital admission. Furthermore, we corroborate prior findings indicating that demographic characteristics and comorbidities are strong mortality predictors in COVID-19 [24, 25]. Interestingly, process mining-related variables such as time decay function values, markings, and token counts were found to have a strong predictive value. These findings advance our understanding of COVID-19 mortality prediction and support further studies using process mining for dynamic risk prediction.

Although previous studies have consistently demonstrated the underlying factors associated with COVID-19 mortality [24], our results highlight those traditional models such as logistic regression or random forest might underestimate the mortality prediction. In contrast to more traditional models, process mining leverages time and the sequence of events. Technically, this was realized through the usage of time functions, which activated the observation of events, and which decayed over time [12]. Multiple types of time decay functions were used, such as linear, exponential, and logarithmic. Each of those functions was initialized based on the mean or maximum patient history duration that was observed in the derivation data set.

By following this approach, predictive models can be developed that update outcome probability based on the time of the prediction. Thus, the likelihood of mortality may change over time, even if no further events have been observed.

The time decay functions values at a given time were fed into a NN, along with event features. Ideally, the NN does not just simply learn the impact of the duration of the last event observation on the outcome probability, but models potentially complex time relationships, such as event interarrival times that have an effect on the outcome probability. These complex time relationships could be the durations between specific lab measurements, or the duration from admission to ICU in the interplay of performed procedures. As clinician behavior may affect event timings and sequencing, the clinician behavior itself may be playing a role in the prediction.

Our results suggest that evaluating the clinical course and the sequence of events up until the time of a prediction can improve predictions as compared to only looking at factors present on admission [25]. Our results help reconcile and summarize findings that demographics, clinical events, laboratory data, and comorbidities can help predict mortality in COVID-19 inpatients. To date, work on artificial intelligence modeling in COVID-19 includes several methodologies, the most frequent being LR, XGBoost, support vector machine, RF, among others [7]. Although current artificial intelligence models have exhibited promising mortality predictive ability, it is unclear which of these methodologies might provide a better prediction compared to others. Moreover, available models do not consider the patient time course in addition to baseline covariates [26, 27]. This is crucial since it can promote early identification of COVID-19 patients with high mortality risk, helping improve clinical decision-making and resource allocation.

At a more general level, our findings are consistent with the concurrent evaluation of the clinical course and available clinical data [24]. Therefore, our work highlights the importance of a comprehensive evaluation of COVID-19 inpatients, including the sequence of clinical events.

A second important finding of this study was the added value of TSS on the process mining model development as time passes, which to date has not been used in COVID-19 prediction models [7]. Based on the results of the Shapley analysis, the time decay function values, and the distinct process mining variables such as markings and token counts, consistently demonstrated an important role in the mortality risk. Hence, our findings underscore the importance of carefully modeling mortality risk while taking into account the series of clinical events among hospitalized COVID-19.

Our approach outperformed other published models in terms of the accuracy, specificity, sensitivity, and AUROC values [13], as well as the best baseline internal model.

### Study limitations

Our results should be interpreted in the light of several limitations. First, our modeling was performed using data from a single site, and these models may have performed differently in other cohorts; as a result, our process should be repeated externally to validate the value of adding time and sequence information in other data sets. Second, our data reflect the first COVID-19 wave in Chicago, therefore, it may not reflect the impact from COVID-19 variants, developed therapies, or vaccination. Third, our dataset contained only a modest number of patients and validation in larger cohorts is needed. Lastly, data validation for report time versus event occurrence time, were demanding, limiting the evaluation of the process mining model in real-time.

## Conclusion

A process mining/deep learning approach using admission data and clinical course of hospitalized COVID-19 patients was able to predict mortality in 6-h intervals within the first 72 h of admission and performed significantly better than the commonly used approach of using only the initial admission results. Our findings underscore the importance of adopting clinical event times and sequencing in the study of COVID-19 mortality, which may help identify underlying characteristics among patients at risk. Since the use of TSS in process mining improved the prediction of COVID-19 mortality, strategies should be considered while identifying those sequential clinical changes, therefore helping to target treatments and resources among those at risk.

There are several avenues for future research. First, the resulting DREAM model can be used to discover if the non-observance of future events (such as action to be performed) has a positive or negative impact on the prediction to facilitate decision making. Such research efforts might enable the detection of improved intervention points in time. Second, sensitivity analyses can be performed to investigate the modeled time dependencies to gain new knowledge about COVID-19 care. This also allows us to investigate the robustness of the model to detect weaknesses that can be further improved. Lastly, our modeling can be used on larger and more diverse datasets and could be continued to be applied as new variants are observed and new vaccines and treatments introduced to assess their impact on clinical outcomes.

## Data Availability

The datasets generated and/or analyzed during the current study are not publicly available due privacy but are available from the corresponding author on reasonable request.

## Appendix

Table 6 shows the variables which were used as inputs to the proposed model. These variables are related to one of the following categories: demographics information, process mining, comorbidities, locations, encounters, procedure reports and the lab measurements. Moreover, where applicable, possible values of the variables are shown.

**Table 6:** Detailed variables were used as inputs to the proposed model

Variables related to specific category	Variables	Variables values (if applicable)
Demographics	Age	
Demographics	Gender	
Demographics	Race	
Process mining	EventCount	
Process mining	TokenCount	
Process mining	Marking	
Process mining	LinearDecay	
Process mining	LinearDecay_mean	
Process mining	ExpDecay_max	
Process mining	LogDecay_mean	
Comorbidities	Hypertension	
Comorbidities	Diabetes	
Comorbidities	Heart Disease	
Comorbidities	COPD	
Comorbidities	Stroke	
Comorbidities	Cerebrovascular Disease	
Comorbidities	Cancer	
Comorbidities	Respiratory Problems	
Comorbidities	Chronic Kidney Disease	
Comorbidities	Tuberculosis	
Location	COVID-4	
Location	COVID-2	
Location	MEDICAL INTENSIVE	
Location	FAMILYMEDICINE	
Location	MICU-2	
Location	MED SERVICE A	
Location	MED SERVICE D	
Location	MED SERVICE C	
Location	MED SERVICE B	
Location	MiCU-1	
Location	MED SERVICE E	
Location	COVID-5	
Location	COVID MICU-3	
Location	MED HEMATOLOGY	
Location	MED HEPATOLOGY/LIVER	
Location	MED SICKLE CELL	
Location	COVID MICU-5	
Location	ORGAN TRANSPLANT	
Location	MED ONCOLOGY	
Location	COVID MICU-4	
Location	STEM CELL TRANSPLANT	
Location	PED PREADMIT ONLY	
Location	COVID-6	
Location	SURGERY GENERAL	
Location	NEUROSURGERY	
Location	MED CARDIO	
Location	CORONARY CARE UNIT	
Location	NEUROLOGY	
Location	MED PREAD ONLY	
Location	MED GI	
Encounters	Inpatient	
Encounters	UIH ER	
Encounters	death	
Encounters	PREADMIT	
Encounters	ER OB	
Encounters	5 W PEDS	
Encounters	disch	
Procedure reports	RADRPT	
Procedure reports	ECG Measurements and Interpretation	
Procedure reports	Echo Transthoracic	
Procedure reports	Pathology Report	
Procedure reports	Echo Transesophageal	
Lab	(1,3)-BETA-D-GLUCAN	Normal
Lab	(1,3)-BETA-D-GLUCAN INTERPRETATION	Normal
Lab	% BASOPHIL	Normal
Lab	% EOSINOPHIL	Normal
Lab	% LYMPHOCYTE	Normal
Lab	% MONOCYTE	Normal
Lab	% NEUTROPHIL	Normal
Lab	% TRANSFERRIN SAT	Normal, LOW, HI
Lab	A. GALACTOMANNAN AG	Normal
Lab	A. GALACTOMANNAN INDEX	Normal
Lab	A1ANTITRYP	Normal
Lab	ABO/RH(D)	No flag
Lab	ABS CD19	Normal, LOW
Lab	ABS CD3	Normal, LOW
Lab	ABS CD3/CD4	LOW
Lab	ABS CD3/CD8	Normal,LOW
Lab	ABS CD56	Normal,LOW,HI
Lab	Abs Retic	Normal,HI
Lab	ABSOLUTE BAND NEUTROPHIL (MANUAL DIFF)	Normal
Lab	ABSOLUTE BASOPHIL (MANUAL DIFF)	HI
Lab	ABSOLUTE EOSINOPHIL (MANUAL DIFF)	Normal, HI
Lab	ABSOLUTE LYMPHOCYTE (MANUAL DIFF)	Normal, LOW, HI
Lab	ABSOLUTE MONOCYTE (MANUAL DIFF)	Normal, LOW, HI
Lab	ABSOLUTE NEUTROPHILS (MANUAL DIFF)	Normal, HI
Lab	ACETAMINOPHEN	LOW
Lab	ACT BICARB	Normal, LOW, HI
Lab	ADAMTS13	LOW
Lab	ADDITIONAL TESTING	Normal
Lab	ADENOVIRUS	Normal
Lab	ADENOVIRUS QUANT BY PCR	Normal
Lab	AEROMONAS/PLEISOMONAS SCREEN	Normal
Lab	ALB CONC	Normal
Lab	ALBUMIN	Normal, LOW
Lab	Alcohol, Urn Screen	Normal
Lab	ALK PHOS	Normal, LOW, HI
Lab	ALT(SGPT)	Normal, LOW, HI
Lab	amd	LOW
Lab	AMMONIA	HI
Lab	AMORPHOUS	Normal
Lab	AMPHETAMINES-UR	Normal
Lab	Amphetamines, Urn Screen	Normal
Lab	AMYLASE	HI
Lab	ANION GAP	Normal, HI
Lab	ANISOCYTOSIS	Normal
Lab	ANTI NUCLEAR AB	Normal
Lab	ANTI-HB CORE IGM	Normal
Lab	ANTI-MITOCHONDRIAL IGG	Normal
Lab	ANTI-SMOOTHMUSCLE	Normal
Lab	ANTIBODY SCREEN	No flag
Lab	ASPERGILLUS AB BY ID	Normal
Lab	AST(SGOT)	Normal, LOW, HI
Lab	ATYPICAL BACTERIAL PNEUMONIA	Normal
Lab	B-NATRIURETIC PEPTIDE	Normal, HI
Lab	BAND NEUTROPHIL	Normal
Lab	BARBITURATES-UR	Normal
Lab	Barbiturates, Urn Screen	Normal
Lab	BASE EXCESS	Normal
Lab	BASO	Normal
Lab	BASOPHILS	Normal, HI
Lab	Benzodiazepines, Urn Screen	Normal
Lab	BENZODIAZPINE-UR	Normal
Lab	BETAHYDROXYBUTYRIC ACID	Normal, HI
Lab	BF ALBUMIN	Normal
Lab	BF BILIRUBIN	Normal
Lab	BF GLUCOSE	Normal
Lab	BF LDH	Normal
Lab	BF LYMPH	Normal
Lab	BF MACROS/MONOS	Normal
Lab	BF MESO	Normal
Lab	BF NEUT	Normal
Lab	BF TOTAL PROTEIN	Normal
Lab	BF-RBC	Normal, HI
Lab	BF-WBC	Normal
Lab	BILIRUBIN, DIRECT	Normal, HI
Lab	BILIRUBIN,TOTAL	Normal, HI
Lab	BKV QUANT BY PCR	Normal
Lab	BKV RT SPECIMEN	Normal
Lab	Blastomyces AB	Normal
Lab	BLASTOMYCES INTERPRETATION	Normal
Lab	BLASTOMYCES RESULT	Normal
Lab	BLASTOMYCES SPECIMEN	Normal
Lab	Bordetella parapertussis	Normal
Lab	BORDETELLA PERTUSSIS	Normal
Lab	BRPR	ABN
Lab	BUDDING YEAST	Normal
Lab	BUN	Normal, LOW, HI
Lab	BUN/CREAT RATIO	Normal, LOW, HI
Lab	BURR CELLS	Normal
Lab	C DIFFICILE RT PCR	Normal
Lab	C-REACTIVE PROTEIN	Normal, HI
Lab	CALCIUM	Normal, LOW, HI
Lab	CALPROTECTIN, FECAL	HI
Lab	CAMPYLOBACTER GROUP BY PCR	Normal
Lab	CARBMAZPNE, UNBOUND	Normal
Lab	CD19%, TOTAL B CELLS	Normal, HI
Lab	CD3/CD4%, HELPER T	Normal, LOW
Lab	CD3/CD8%, SUP T CELLS	Normal, HI
Lab	CD3%, TOTAL T CELLS	Normal, LOW
Lab	CD4 COMMENT	Normal
Lab	CD56%	Normal, HI
Lab	CDASU 9A Comments	Normal
Lab	CEA	HI
Lab	CERULOPLASMIN	LOW
Lab	CHK	No flag
Lab	CHLAMYDIA PNEUMONIAE	Normal
Lab	CHLORIDE	Normal, LOW, HI
Lab	CHOLESTEROL	Normal, HI
Lab	CK MACRO TYPE I	Normal
Lab	CK MACRO TYPE II	Normal
Lab	CK TOTAL	Normal
Lab	CK-BB	Normal
Lab	CK-MB	Normal
Lab	CK-MM	Normal
Lab	CLARITY	Normal
Lab	CLUMPED PLATELETS	Normal
Lab	CMV QUANT BY PCR	Normal
Lab	CO2 CONTENT	Normal, LOW, HI
Lab	COCAINE-URINE	Normal
Lab	Cocaine, Urn Screen	Normal
Lab	COLOR	Normal
Lab	COMPLEMENT C3	LOW
Lab	COMPLEMENT C4	Normal
Lab	COPPER	HI
Lab	Coronavirus 19	Normal, ABN
Lab	CORONAVIRUS 229E	Normal
Lab	CORONAVIRUS HKU1	Normal
Lab	CORONAVIRUS NL63	Normal
Lab	CORONAVIRUS OC43	Normal
Lab	CPK	Normal, LOW, HI
Lab	CREAT CONC	Normal
Lab	CREATININE	Normal, LOW, HI
Lab	Creatinine, Urn Screen	Normal
Lab	CROSSMATCH	No flag
Lab	CYTOPLASMIC STAINING	Normal
Lab	D-DIMER	Normal, HI, CRIT
Lab	DIFF METHOD	Normal
Lab	DIFFERENTIAL METHOD	Normal
Lab	DOHLE BODIES	Normal
Lab	EBV QUANT BY PCR	Normal, ABN
Lab	EOS	Normal, HI
Lab	EOSINOPHIL	Normal, HI
Lab	Estimated Creat Clearance	No flag, LOW
Lab	Estimated GFR	No flag
Lab	ETHANOL	Normal
Lab	FENTANYL QUANT URINE	Normal
Lab	FERRITIN	Normal, LOW, HI
Lab	FIBRINOGEN	Normal, HI
Lab	FINE GRAN CAST	HI
Lab	FK506/TACROLIMUS	Normal
Lab	Flu A (POCT)	Normal
Lab	FLU A H1 SEASONAL	Normal
Lab	FLU A H1N1 2009	Normal, ABN
Lab	FLU B	Normal
Lab	Flu B (POCT)	Normal
Lab	FOLATE	Normal
Lab	FREE T4	Normal, LOW
Lab	GLUCOSE	Normal, LOW, HI, CRIT
Lab	GLUCOSE (POCT)	Normal, LOW, HI, CRIT
Lab	HAPTOGLOBIN	Normal, HI
Lab	HCT	Normal, LOW, HI
Lab	HCV REAL TIME PCR	Normal
Lab	HDL	Normal, LOW
Lab	HELP/SUPP RATIO	Normal
Lab	Hemoglobin—POCT	LOW
Lab	HEMOGLOBIN A2	Normal
Lab	HEMOGLOBIN F	Normal, HI
Lab	HEP A IGM AB	Normal
Lab	HEP B CORE AB,TOTAL	Normal
Lab	HEP B SURF AB,QUANT	Normal
Lab	HEP B SURFACE AG	Normal
Lab	HEP C ANTIBODY	Normal, ABN
Lab	HGB	Normal, LOW, HI
Lab	HGB A	Normal
Lab	HGB A1C	Normal, HI
Lab	HGB C	Normal
Lab	HGB S	Normal
Lab	HISTOPLASMA INTERPRETATION	Normal
Lab	HISTOPLASMA RESULT	Normal
Lab	HISTOPLASMA SPECIMEN	Normal
Lab	HIV 1 Antibody	Normal
Lab	HIV 1 Antigen	Normal
Lab	HIV 2 Antibody	Normal
Lab	HIV Antigen and Antibody Screen NC	Normal
Lab	HIV1AB	Normal
Lab	HIV1AG	Normal
Lab	HIV2AB	Normal
Lab	HOWELL JOLLY	Normal
Lab	HSV TYPE I	Normal
Lab	HSV TYPE II	Normal
Lab	HUMAN METAPNEUMOVIRUS	Normal
Lab	HUMAN RHINOVIRUS/ENTEROVIRUS	Normal
Lab	HVABAG	Normal
Lab	HYALINE CAST	Normal
Lab	HYPOCHROMASIA	Normal
Lab	IGA	Normal, LOW, HI
Lab	IGG	Normal, LOW
Lab	IGM	Normal, LOW, HI
Lab	IMMUNOFIX SERUM	Normal
Lab	Influenza A Equivocal (Inconclusive)	Normal
Lab	INFLUENZA A, H3 SUBTYPE	Normal
Lab	Influenza A, No Subtype Detected	Normal
Lab	INR	Normal, HI, CRIT
Lab	INTERLEUKIN 6	Normal, HI
Lab	INTERPRETATION	Normal
Lab	IONIZED CALCIUM	Normal, LOW
Lab	IRON	Normal, LOW, HI
Lab	Issue Date/Time	No flag
Lab	LACTIC ACID	Normal, LOW, HI, CRIT
Lab	LARGE PLATELETS	Normal
Lab	LDH	Normal, HI
Lab	LDL, CALCULATED	Normal, HI
Lab	LEGIONELLA AG, UR	Normal
Lab	LEUK ESTERASE	Normal, ABN
Lab	LEVETIRACETAM LEVEL	LOW
Lab	LIPASE	Normal, LOW, HI
Lab	LITHIUM	Normal
Lab	LYMPH	Normal, LOW, HI
Lab	LYMPHOCYTE	Normal, LOW, HI
Lab	MACROCYTOSIS	Normal
Lab	MAGNESIUM	Normal, LOW ,HI
Lab	MARIJUANA-URINE	Normal, ABN
Lab	Marijuana, Urn Screen (THC, Urn, Screen)	Normal
Lab	MCH	Normal, LOW, HI
Lab	MCHC	Normal, LOW
Lab	MCV	Normal, LOW, HI
Lab	MEAS O2 SAT-MV	Normal, LOW, HI
Lab	META	HI
Lab	Methadone, Urn Screen	Normal
Lab	METHANOL	Normal
Lab	MICROALB/CREAT RATIO	HI
Lab	MICROCYTOSIS	Normal
Lab	MITOGEN MINUS NIL	Normal
Lab	MONO	Normal, LOW, HI
Lab	MONOCYTE	Normal, LOW, HI
Lab	MPV	Normal, LOW, HI
Lab	MRSA Transcribed Result	No flag
Lab	MUCUS	Normal
Lab	MYELO	HI
Lab	NEUT	Normal, LOW, HI
Lab	NEUTROPHIL	Normal, LOW, HI
Lab	NIL (NEGATIVE CONTROL)	Normal
Lab	NITRITE	Normal, ABN
Lab	NON FENTANYL URINE	Normal
Lab	Non-HDL Chol	No flag
Lab	NOROVIRUS GI/GII BY PCR	Normal
Lab	NUCLEATED RBC'S	Normal
Lab	O2 SAT	Normal, LOW, HI
Lab	O2 SAT MEASURED	Normal, LOW
Lab	OPIATE HYDROCODONE	Normal
Lab	OPIATE ACETYL MORPHINE	Normal
Lab	OPIATE CODEINE	Normal
Lab	OPIATE HYDROMORPHONE	Normal
Lab	OPIATE MORPHINE	Normal
Lab	OPIATE OXYCODONE	Normal
Lab	OPIATE OXYMORPHONE	Normal
Lab	OPIATES NORHYDROCODONE	Normal
Lab	OPIATES NOROXYCODONE	Normal
Lab	OPIATES NOROXYMORPHONE	Normal
Lab	OPIATES-URINE	Normal, ABN
Lab	Opiates, Urn Screen	Normal
Lab	OVA AND PARASITES EXAM	Normal
Lab	OVALOCYTES	Normal
Lab	PARA1	Normal
Lab	PARA2	Normal
Lab	PARA3	Normal
Lab	PARA4	Normal
Lab	PARVOVIRUS QUANT BY PCR	Normal
Lab	PCO2	Normal, LOW, HI, CRIT
Lab	PCT FREE CARB	Normal
Lab	PERFORMING LAB	Normal
Lab	PH	Normal, LOW, HI
Lab	PHENCYCLIDINE UR	Normal
Lab	Phencyclidine, Urn Screen	Normal
Lab	PHENYTOIN FREE	Normal
Lab	PHENYTOIN TOTAL	Normal, LOW
Lab	PHOSPHORUS	Normal, LOW, HI, CRIT
Lab	PLT	Normal, LOW, HI, CRIT
Lab	PLT ESTIMATE	Normal
Lab	PO2	Normal, LOW, HI
Lab	POIKILOCYTOSIS	Normal
Lab	POLYCHROMASIA	Normal
Lab	POTASSIUM	Normal, LOW,HI, CRIT
Lab	PRO BNP,NT	Normal, HI
Lab	PROCALCITONIN	Normal
Lab	Product Code	No flag
Lab	Product Identification	No flag
Lab	PROLACTIN	Normal
Lab	Propoxyphene, Urn Screen	Normal
Lab	PROT/CREAT RATIO	Normal
Lab	PROTHROMBIN TIME	Normal, HI
Lab	PTH-INTACT	HI
Lab	PTT	Normal, LOW, HI, CRIT
Lab	QTBG INTERPRETATION	Normal
Lab	QUANTIFERON TB RESULT	Normal
Lab	RBC	Normal, LOW, HI
Lab	RDW	Normal, HI
Lab	REACTIVE LYMPHS	Normal
Lab	RESPIRATORY PCR PANEL SPECIMEN SOURCE	Normal
Lab	RESPIRATORY SYNCYTIAL VIRUS	Normal
Lab	RETIC COUNT	Normal, HI
Lab	ROTAVIRUS A BY PCR	Normal
Lab	SALICYLATE	Normal
Lab	SALMONELLA SPECIES BY PCR	Normal
Lab	SARS-CoV-2 IGG AB	Normal, ABN
Lab	SCHISTOCYTES	Normal
Lab	SED RATE-WEST	Normal, HI
Lab	SEND OUT RESULT:	Normal
Lab	SEND OUT TEST:	Normal
Lab	SERUM ALB ELECT	Normal
Lab	SERUM ALPHA 1	Normal
Lab	SERUM ALPHA 2	Normal
Lab	SERUM BETA	Normal
Lab	SERUM GAMMA	Normal
Lab	SERUM HCG	Normal
Lab	SERUM OSMOLALITY	Normal, LOW, HI, CRIT
Lab	SERUM TOTAL PROTEIN	Normal
Lab	SFIX ENHANCED REPORT	Normal
Lab	SHIGA TOXIN 1 BY PCR	Normal
Lab	SHIGA TOXIN 2 BY PCR	Normal
Lab	SHIGELLA SPECIES BY PCR	Normal
Lab	SICKLE CELLS	Normal
Lab	SODIUM	Normal, LOW, HI
Lab	SPECIMEN SOURCE	Normal
Lab	SPECIMEN TYPE	Normal
Lab	SPHEROCYTES	Normal
Lab	SQUAMOUS EPI'S	Normal, HI
Lab	Status Information	No flag
Lab	STREPTOCOCCUS PNEUMONIAE AG, URINE	Normal
Lab	SYPHILIS FOLLOW UP, RPR QUANT	Normal
Lab	TARGET CELLS	Normal
Lab	TB AG MINUS NIL	Normal
Lab	TB SCR COMMENT	Normal
Lab	TB2 AG MINUS NIL	Normal
Lab	TEARDROPS	Normal
Lab	TOTAL CARB	Normal
Lab	TOTAL IRON BINDING	Normal, LOW, HI
Lab	TOTAL PROTEIN	Normal, LOW, HI
Lab	Total Syphilis Antibody IGG and IGM	ABN
Lab	TOXIC VACUOLIZATION	Normal
Lab	TRANS EPI CELLS	Normal, HI
Lab	TRANSFERRIN	Normal, LOW
Lab	Treponema pallidum Antibody by TP-PA	Normal
Lab	TRIGLYCERIDE	Normal, HI
Lab	TROPONIN I	Normal, HI, CRIT
Lab	TSH	Normal, LOW, HI
Lab	Unit Blood Type	No flag
Lab	Unit Number	No flag
Lab	UR CHLORIDE-RANDOM	Normal
Lab	UR CREATININE	Normal
Lab	UR OSMOLALITY	Normal, LOW, HI
Lab	UR PH	Normal
Lab	UR POTASSIUM-RANDOM	Normal
Lab	UR SODIUM-RANDOM	Normal
Lab	UR TOTAL PROTEIN	Normal
Lab	UR UREA N-RANDOM	Normal
Lab	URIC ACID	Normal, LOW, HI
Lab	Urine bacteria	ABN
Lab	URINE BILIRUB	Normal
Lab	URINE BLOOD	Normal,ABN
Lab	URINE CLARITY	Normal
Lab	URINE COLOR	Normal
Lab	URINE GLUCOSE	Normal,ABN
Lab	URINE HCG	Normal
Lab	URINE KETONES	Normal,ABN
Lab	Urine pregnancy test—POCT	No flag
Lab	URINE PROTEIN	Normal,ABN
Lab	Urine RBC's	Normal,HI
Lab	URINE SP GRAV	Normal,HI
Lab	Urine WBC's	Normal,HI
Lab	UROBILINOGEN	Normal,HI
Lab	VANCOMYCIN-RANDOM	Normal
Lab	VIBRIO GROUP BY PCR	Normal
Lab	VITAMIN B1	Normal
Lab	VITAMIN B12	Normal,HI
Lab	VITAMIN D (25 OH)	LOW
Lab	Volume	No flag
Lab	WAXY CAST	Normal
Lab	WBC	Normal,LOW,HI
Lab	WBC CLUMPS	Normal
Lab	WHOLE BLOOD GLUC	Normal,HI,CRIT
Lab	WHOLE BLOOD HGB	Normal,LOW
Lab	WHOLE BLOOD K	Normal,LOW,HI,CRIT
Lab	WHOLE BLOOD NA	Normal,LOW,HI
Lab	YERSINIA ENTEROCOLITICA BY PCR	Normal
Lab	ZINC, BLOOD	Normal
Vit	BMI	ok
Vit	BP diastolic	ok
Vit	BP systolic	ok
Vit	Pulse rate	ok
Vit	Respiratory rate	ok
Vit	SPO2	ok,crit
Vit	Temp (DegC)	ok,crit

## Abbreviations

AUROC: Average area under the receiver operating characteristic curve
AI: Artificial intelligence
COVID-19: Coronavirus disease 2019
DREAM: Decay replay mining
LR: Logistic regression
NN: Neural network
RF: Random forest
TSS: Timed state sample
LDH: Lactate dehydrogenase
CRP: C-reactive protein
